# Analysis of a Hybrid Micro-Electro-Mechanical Sensor Based on Graphene Oxide/Polyvinyl Alcohol for Humidity Measurements †

**DOI:** 10.3390/s19071720

**Published:** 2019-04-10

**Authors:** Carlo Trigona, Ammar Al-Hamry, Olfa Kanoun, Salvatore Baglio

**Affiliations:** 1 DIEEI, Dipartimento di Ingegneria Elettrica, Elettronica e Informatica, University of Catania, 95125 Catania, Italy; salvatore.baglio@unict.it; 2 Professorship of Measurement and Sensor Technology, Chemnitz University of Technology, ReichenhainerStraße 70, 09126 Chemnitz, Germany; ammar.al-hamry@etit.tu-chemnitz.de (A.A.-H.); olfa.kanoun@etit.tu-chemnitz.de (O.K.)

**Keywords:** redundant measurements, hybrid sensor, nanomaterial, GO/PVA composite, humidity sensor, MEMS

## Abstract

In this paper, we present a redundant microsensor based on the bulk and etch silicon‑on‑insulator (BESOI) process for measuring relative humidity (RH), by using a graphene‑oxide/polyvinyl‑alcohol (GO/PVA) composite. The MEMS is a mechanical oscillator, composed of a proof mass with multilayer of nanomaterials (GO/PVA) and suspended by four crab-leg springs. The redundant approach realized concerns the use of different readout strategies in order to estimate the same measurand RH. This is an intriguing solution to realize a robust measurement system with multiple outputs, by using the GO/PVA as functional material. In the presence of RH variation, GO/PVA (1) changes its mass, and as consequence, a variation of the natural frequency of the integrated oscillator can be observed; and (2) varies its conductivity, which can be measured by using two integrated electrodes. The sensor was designed, analyzed and modeled; experimental results are reported here to demonstrate the effectiveness of our implementation.

## 1. Introduction

Recently, extensive research has been conducted on the design and conception of smart solutions for measurement systems, as well as robust/redundant strategies based on multiple transducers and repeated sensing elements [1,2]. The main purposes of redundant approaches presented in literature are to provide highly reliable and accurate measurements [2].

In this context, the interest of the scientific community is highly felt for macro-scale devices, but also in small and integrated scale sensors (MEMS) [3]. The redundant approaches mentioned are in regards to the use of several/multiple sensors to compare the measurement. This implies a considerable number of devices/nodes to be used and compared.

Humidity sensors have earned considerable attention, due to the increasing demand for moisture monitoring in semiconductor industries, automobile industries, living environments and domestic applications [4]. Humidity sensors are realized considering different readouts, such as capacitive, piezoresistive, optical, piezoelectric and quartz-based transduction and optical and glass-fiber architectures [5].

There are also some high-precision, impedance-frequency transducers using quartz crystals, which compensate for temperature drift and have a fast response. At high air humidity measurements, there is a problem with response time of the sensors using conventional methods. The solution for this problem is a sensor for high air humidity measurements, which uses an open capacitor with very low response time [6,7,8,9].

It is worth noting that the polymer Polyaniline (PANI) and PVA [10], carbon nanotubes [11], or graphene oxide-based [12,13] humidity sensors have already been proposed in literature. In particular, polymer nanocomposites could bring low-cost processing, both resistive and capacitive responses and a wide range of humidity sensing. Polymer nanocomposites having filler that is at least one dimension in nanoscale have brought considerable attention for investigation, since Kojima et al. [14] at first reported nylon/clay nanocomposites. They are often recognized for tuned thermal, electrical and mechanical properties different from their parent materials. The functional groups of graphene oxide (GO) add the dispersibility inside the polyvinyl alcohol (PVA) matrix or other polar aqueous media. Kashyap et al. [15] found ~150% elastic modulus increment after 0.3 weight (wt.)% loading in PVA. The change in the property was believed to be happening due to the strong interfacial interaction between filler and matrix. Elastic modulus, strength and electrical conductivity depend on the distribution of fillers and degree of interfacial interactions. Exfoliation of GO by different methods, i.e., thermal and sonication, produces flakes of different lateral dimensions and the size of those flakes is a key determinant of interfacial interactions and mechanical interlocking. Another important aspect of GO in polymer nanocomposites is that with very low filler loading, improved properties can be obtained. Wang et al. [16] found a percolation network in a polypropylene matrix with 0.033 volume (vol.)% of GO filler. In [17], authors found percolation with 0.5 wt.% of GO filler in a PVA matrix with conductivity of about 8 × 10^−6^ S/cm.

GO is electrically insulating, due to loss of *π* bonds, but a percolated pathway realized inside the matrix and a successive chemical or thermal reduction will increase the conductivity by a partial restoration of the *sp*^2^ network. Along with the change in electrical characteristics, nano-sized fillers inside the matrix will also alter the permittivity and enhance its charge storing capability. Due to the high aspect ratio of the fillers, the composite exhibits a higher dielectric constant at a lower loading [18]. PVA, on the other hand, is a widely used thermoplastic, water soluble and nontoxic polymer [19]. PVA is known as a good host for nanocomposites, because of its good thermal stability and chemical resistance. PVA is a synthetic, semi-crystalline, biodegradable and hydrophilic polymer. The carbon backbone of PVA contains hydroxyl groups, which are good sources of hydrogen bonding. PVA enhances the thermal and mechanical stability of the composite at low loading of GO and makes it worthy for many practical applications. PVA was categorized as polyelectrolyte in nature, and by creating porous thin films with PVA it is possible to exploit humidity sensing property out of the material. Chen et al. [4] have found PVA to be sensitive to more than 50% RH. However, the properties of the nanocomposite avail the opportunity to deposit it as a thin film of different thicknesses and then use it as a capacitive or resistive humidity sensor. Water molecules are accommodated within the GO interlayers, hence the resistive property changes. By selecting the right thickness, a continuous change in resistance and capacitance with respect to humidity covering a large range, i.e., 20% to 80% RH, has been realized [20].

In particular, in this latter paper, the authors present a preliminary study of an integrated hybrid micro sensor based on GO/PVA for humidity measurements; a comprehensive analysis of the device with an accurate description of the design, fabrication, materials analysis, modeling, simulations and nonlinear effects of the MEMS with GO/PVA is presented. Finally, an extensive experimental campaign has been accomplished, and the results obtained that are reported here confirm the suitability of the proposed approach, based on a single device to measure RH by using different readout strategies. 

The method pursued here will be based on the adoption of a GO/PVA composite [21] used as a functional material and deposited over a BESOI-based microsensor [22,23] operating as a mechanical oscillator which designed through MEMSPRO (version 2017). In the presence of an increment of RH, this material is able to increase its inertial mass, and at the same time, it is able to increase its conductivity. At the moment, to our knowledge, this is the first solution of a redundant sensor realized in integrated scale and based on GO/PVA used as a functional layer. The paper is specifically composed of four other sections. In Section 2, the material composition will be described, as well as the working principle of the conceived device. In Section 3, the fabrication technology will be addressed, including the foundry process and the modeling of the sensor. Section 4 presents the experiments conducted and Section 5 concludes the paper.

## 2. Materials and Methods

**Figure 1:**
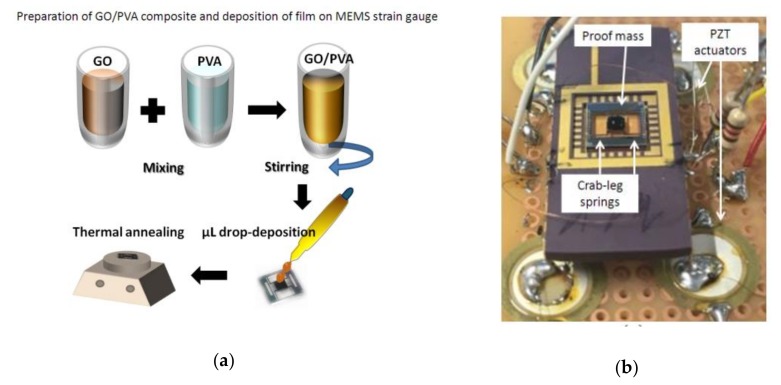
Sensor fabrication: (**a**) graphene‑oxide/polyvinyl‑alcohol (GO/PVA) composite preparation by solution mixing and deposition on the micromachined sensor and (**b**) the realized microsensor and four PZT elements used to move the device.

Aqueous GO solution of 0.5 wt.% (flake size 0.5 to 5 microns) was purchased from Graphene Supermarket. PVA (Sigma-Aldrich CAS Number 9002-89-5; Mw 85,000–124,000) powder was added into water to make 4 wt.% aqueous solution. PVA solution was kept at 90 °C for 2 h, along with magnetic stirring for achieving good solubility. Later, GO solution was poured into the PVA polymer, stirred for 30 min and sonicated for 10 more minutes, (see Figure 1a). Different loading ratios have been considered for GO:PVA (25, 50, and 75%) and the I-V characteristic was tested by making thin film for each loading ratio, in order to test the conductivity. Then, 10 µL was deposited on an area of 5 × 10 mm on glass substrate, after which it was annealed at 300 °C for 30 min. The measurement of IV curves were carried out using a Keithly 2636 source meter interfaced with the LabVIEW program to sweep voltage from −5 to 5 V, with increments of 0.1 V. In addition, to check the morphology and surface properties of the deposited films, scanning electron microscopy (SEM) was carried out with an FEI Nova NanoSEM 200. The samples were prepared by casting the composites on silicon wafers and annealing them at 300 °C and then SEM imaging was performed. Attention was focused on the micromachined sensor, which is composed of four crab-leg beams with a suspended mass of bulk‑silicon and a metal plate is used to contact the GO/PVA located on the proof mass (see Figure 1b). By evaluating the resistance range and the micro-drop deposition possibility, a loading ratio for GO:PVA of 50% was selected for application in dual sensing, as demonstrated in Section 4. An amount of 10 µm was casted on the suspended silicon and dried overnight. In order to increase the conductivity of the GO/PVA sensor, the device was annealed at 300 °C. More details concerning the fabrication of the microsensor will be addressed in the next section.

**Figure 2:**
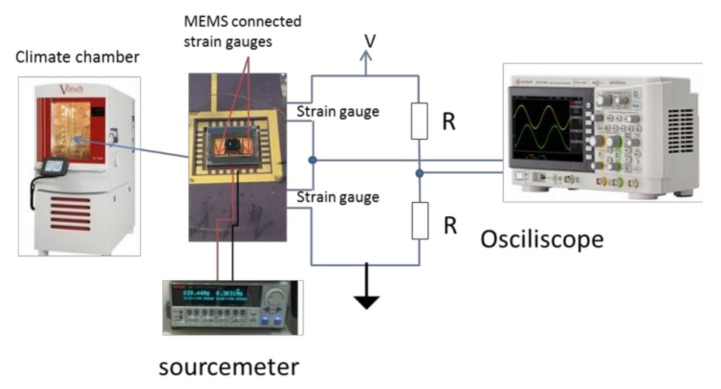
Experimental setup, composed of a humidity chamber to control the humidity and temperature applied to the MEMS. The output was registered by a sourcemeter and an oscilloscope, to obtain the resistance of the GO/PVA film and the oscillation frequency of the bridge, respectively. The voltage V is a DC voltage of ~5 V, in order to have the maximum sensitivity. The configuration of the bridge is a full bridge; in particular, two discrete resistors and two integrated strain gauges (embedded in two crab-leg springs) have been used, respectively.

In order to validate the readout, based on the variation of the natural frequency of the mechanical oscillator as a function of the variation of RH, two integrated strain gauges were connected in a Wheatstone bridge and the output was acquired by using an oscilloscope (LeCroy waverunner 6050), as shown in Figure 2. Four piezoelectric elements (PZTs) have been used to mechanically excite the MEMS. Furthermore, a sourcemeter (Keithley-model 2636) was used to measure the variation of resistance of the functional material connected by using two integrated electrodes.

## 3. Fabrication of the Microsensor with Graphene‑Oxide/Polyvinyl‑Alcohol

**Figure 3:**
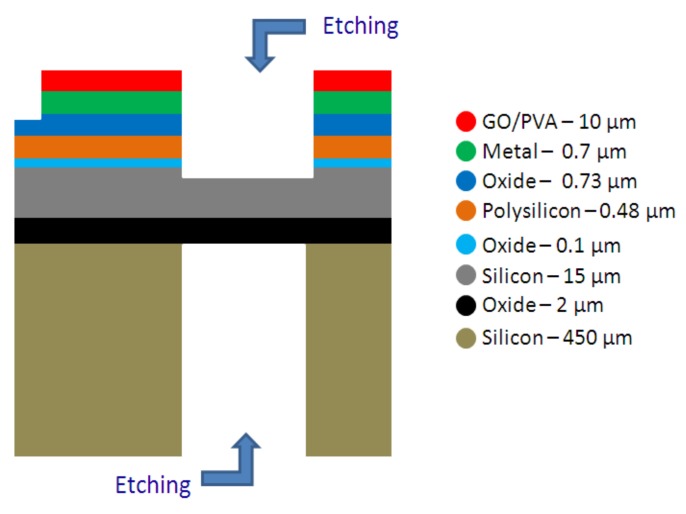
Fabrication process (bulk and etch silicon‑on‑insulator; BESOI) for the realization of the microsensor (not to scale).

The hybrid micro-electro-mechanical sensor, based on GO/PVA, is a suspended mass supported by four crab-leg beams, realized by using a custom BESOI (bulk and etch silicon-on-insulator) process, as shown in Figure 3. The proof mass was realized through a silicon-on-insulator (SOI) substrate, based on 15 μm of doped silicon layer and 450 μm of silicon substrate, with 2 μm of buried oxide. The SOI wafer was processed with a front- and back-side DRIE etching technique. In order to implement a resistive readout based on integrated strain gauges, a polysilicon layer was used. Furthermore, in order to create electrical contacts, a metal layer (of aluminum) with a thickness of 0.7 µm was used. A final layer of GO/PVA was used as a sensing layer for the RH estimation.

**Figure 4:**
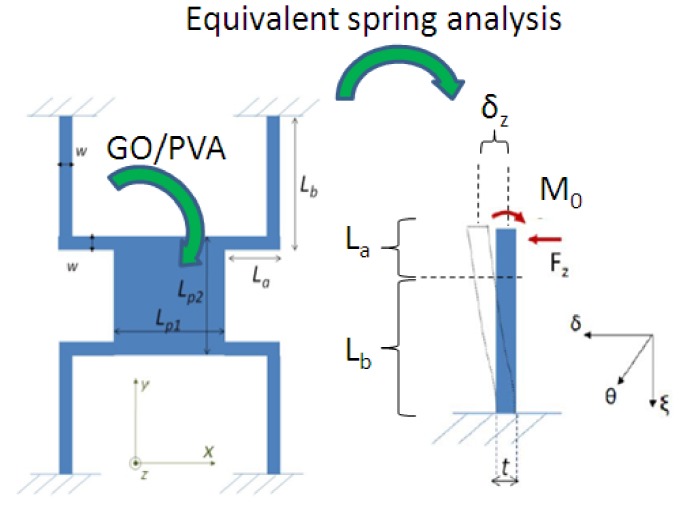
Schematic of the passive microresonator. The axes *δ*, *ξ* and *θ* have been considered to study a single spring. By using the symmetry of the structure, the response of the entire device has been modeled, with its easy axis along the *z* direction.

A reference microresonator device is presented in Figure 4. In particular, the system conceived is composed of a suspended mass of 1600 μm (L_p1_) × 1600 μm (L_p2_) × 450 μm, supported by four crab-leg silicon springs with a width (*w*) of 100 μm and length of 800 μm for the segment (*L_a_*) anchored to the central proof mass and 1600 μm for the segment anchored to the substrate (*L_b_*), (see Figure 4). The microsensor has been designed taking into the account the modeling described in the following.

Under the hypotheses of uniform structure, symmetric geometry, small displacement oscillations and neglecting torsions between the two elements of each spring (*L_a_* and *L_b_*), the strain energy (*U*) of a single arm (in particular, an equivalent spring having a length *L* of *L_a_* + *L_b_* is considered here) can be found through the integration along the beam (*ξ*) (see Figure 4):

(1) $${\left.U=\int_{0}^{L=L_{a}+L_{b}}\frac{M^{2}}{2E_{si}I}d\xi \right|}_{I=\frac{tw^{3}}{12}}$$

where *E_si_* is the Young’s modulus of the silicon and *I* is the bending moment of inertia along the axis of movement and considering a rectangular shape of the spring. In addition, *t* represents the thickness of the silicon structure (15 µm) and *w* the width of the beam (100 µm).

*M* is the beam bending moment, which can be expressed, in accordance with the Figure 4, as the following:

(2) $$M=M_{0}-F_{z}\xi$$

where *F_z_* is the applied force, which will cause a displacement variation (*δ_z_*). The method used to evaluate the elastic constant of the device, and as a consequence, its natural frequency, is based on the application of Castigliano’s theorem [24]. This method requires calculation of the derivative of the strain energy density with respect to the initial moment (*M_0_*), which corresponds to *θ_0_* (the initial angle at the end of the beam) and with respect to the imposed force (*F_z_*), which corresponds to *δ_z_*:

(3) $$\theta_{0}=\frac{\partial U}{\partial M_{0}}=\int_{0}^{L}\frac{M}{E_{si}I}\frac{\partial M}{\partial M_{0}}d\xi$$

Imposing the condition that the initial angle is equal to zero, the equation can be re-written as

(4) $$\theta_{0}=\frac{1}{E_{si}I}M_{0}L-\frac{1}{E_{si}I}F_{x}\frac{L^{2}}{2}=0$$

From Equation (4), it is possible to evaluate the initial moment *M_0_*, and as consequence, the moment *M* can be expressed as

(5) $$M=F_{z}\left(\frac{L}{2}-\xi \right)$$

By applying the theorem of Castigliano a second time, in order to determine the deflection at the end of the beam (δ_z_), the following equation is obtained:

(6) $$\delta_{z}=\frac{\partial U}{\partial F_{z}}=\overset{L}{\underset{0}{\int }}\frac{M}{E_{si}I}\frac{\partial M}{\partial F_{z}}d\xi =\frac{F_{z}L^{3}}{12E_{si}I}$$

The elastic constant, considering this latter equation, and the contribution of all the four springs, can be written as

(7) $$k_{z}=\frac{4F_{z}}{\frac{F_{z}L^{3}}{12E_{si}I}}=\frac{48E_{si}I}{L^{3}}$$

**Figure 5:**
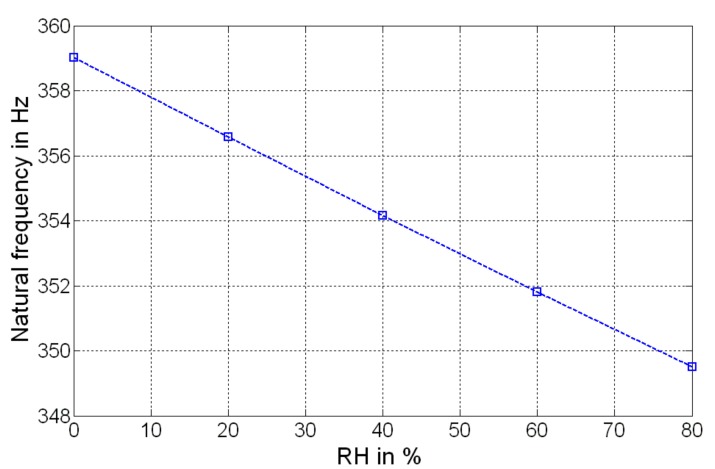
Simulated result: natural frequency as function of the relative humidity (RH).

The natural frequency, considering also the presence of the GO/PVA, can be expressed as

(8) $$f_{n}=\frac{1}{2\pi }\sqrt{\psi \;\frac{k_{z}}{M+\Delta M}}$$

where *ψ* is a multiplicative coefficient that takes into account the effect of the post-processing deposition of the GO/PVA layer. It is worth noting that Δ*M* is the contribution of RH, which increases the proof mass of the MEMS, and as consequence, will cause a decrement of its natural frequency. Considering the values reported in Table 1, a simulation of the frequency shift as a function of RH has been accomplished, as shown in Figure 5.

## 4. Experimental Results

**Figure 6:**
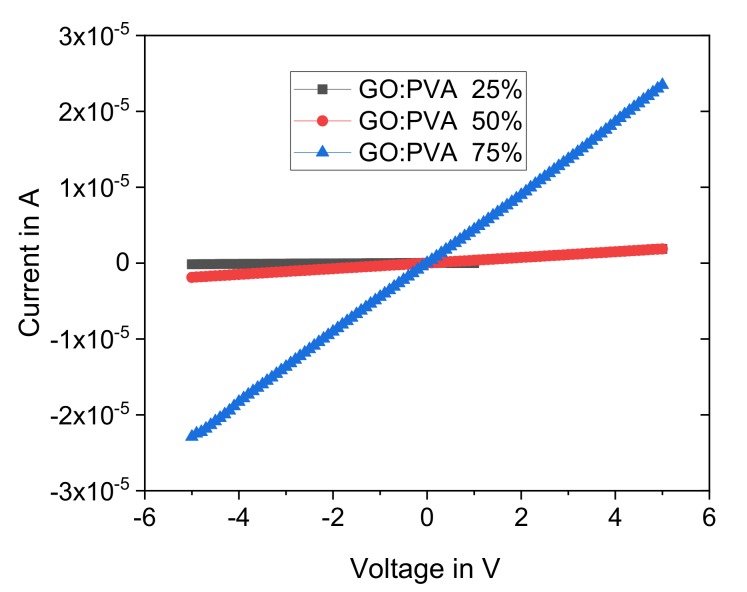
I-V measurement of GO/PVA with different ratios, deposited on glass substrate and annealed at 300 °C. The measurements have been conducted by using a step of 0.1 V. The condition of GO:PVA at 50% was used for the realization of the layer on top of the proof mass of the sensor.

In this section, the results achieved by using the device described in the previous section and through the adoption of the setup presented in Section 2 are presented. In particular, the GO/PVA has been characterized and the choice of the final layer used (GO:PVA = 50%) is also correlated with this analysis. Figure 6 shows the IV characteristic of thin films of GO:PVA deposited by drop casting. From the current versus voltage graphs shown in Figure 6, the resistance decreases (~31.7 MΩ for 25% loading) by several orders of magnitude to reach ~2.6 MΩ and ~225.8 kΩ, for 50% and 75% loading, respectively. Therefore, it can be observed that decreasing the value of percentage RH, from 75% to 50%, does not provide a significant improvement in terms of a high value of resistance.

**Figure 7:**
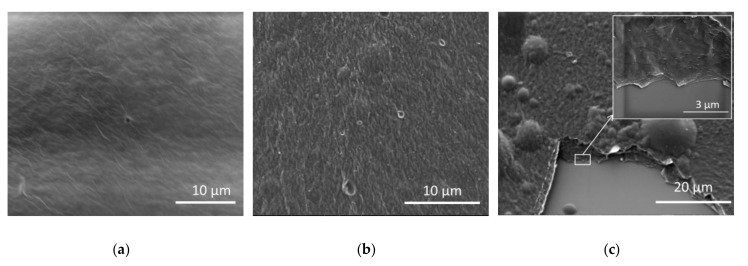
Scanning electron microscopy (SEM) images of GO/PVA at different mixing ratios: (**a**) 25%, (**b**) 50% and (**c**) 75%. The inset in (**c**) shows a cross-section, where stacked layer of GO are well-embedded in PVA polymer. Aggregation is mainly correlated with the nature of the drop-casting method, especially for a high percentage of GO, while for lower concentrations a more regular distribution can be observed.

SEM images are shown in Figure 7, in which 10 microliters of GO/PVA with the different ratios were deposited on a Si wafer. The morphology of the composites indicates that GO is well-dispersed within the PVA polymer. The existence of aggregation is due to the nature of the drop casting deposition. The homogeneity of the GO stacked layer embedded in the polymer matrix, even with high loading (75%), is noticeable (see Figure 7c inset). However, because of the annealing process for the reduction of GO, the swelling and break of deposited films is observed at a high percentage of GO. It was found that the GO:PVA ratio of 50% gives both mechanical stability and good electrical conductivity, in comparison to other ratios. At a low ratio, the mechanical stability and integrity of the films after annealing is good, but the electrical conductivity is poor. At a higher ratio, the mechanical properties become worse, because at this annealing temperature the films show breaks and porous structures because of loss of water and gases during reduction by the annealing process [25]. For this reason, the best tradeoff, in order to have a high value of resistance with a simpler detection of its variation as function of the RH, is represented by 50% of GO:PVA.

**Figure 8:**
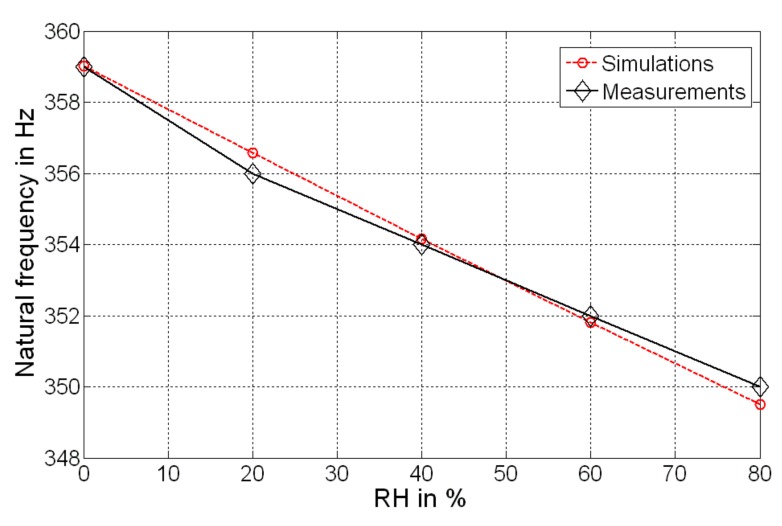
Natural frequency shift as a function of the measurand. A comparison with respect to the simulation through Equation (8) is accomplished.

**Figure 9:**
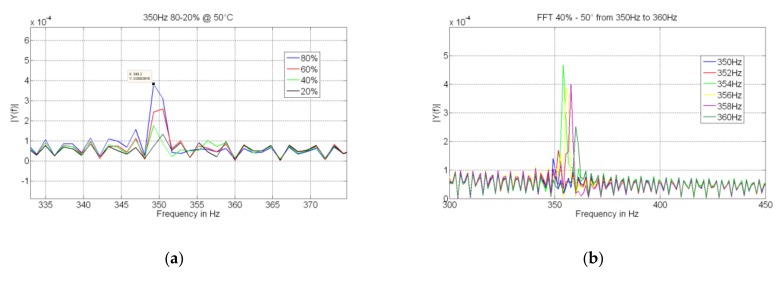
(**a**) FFT of the output of the bridge at 350 Hz (50 °C); (**b**) analysis around the mechanical resonance (at 40%, 50 °C).

The mechanical response of the microsensor has been experimentally validated through the estimation of its natural frequency shift, as a function of the RH absorbed by the GO/PVA layer. Figure 8 shows the frequency variation compared with the model implementation presented in Section 3. Good agreement between both methods can be seen. A more exhaustive analysis can be shown in Figure 9a, where it shows the FFT of the output of the bridge for a fixed mechanical frequency (350 Hz), imposed through four piezoelectric actuators. The result shows the effect of the spike for several RH amplitudes (from 20% to 80%). A decrement can be observed as a consequence of the movement of the natural frequency of the oscillator. It is worth mentioning that in order to validate the resonance, a variation in terms of excitation frequency has been implemented, and as shown in Figure 9b, a maximum spike can be observed in resonant condition.

**Figure 10:**
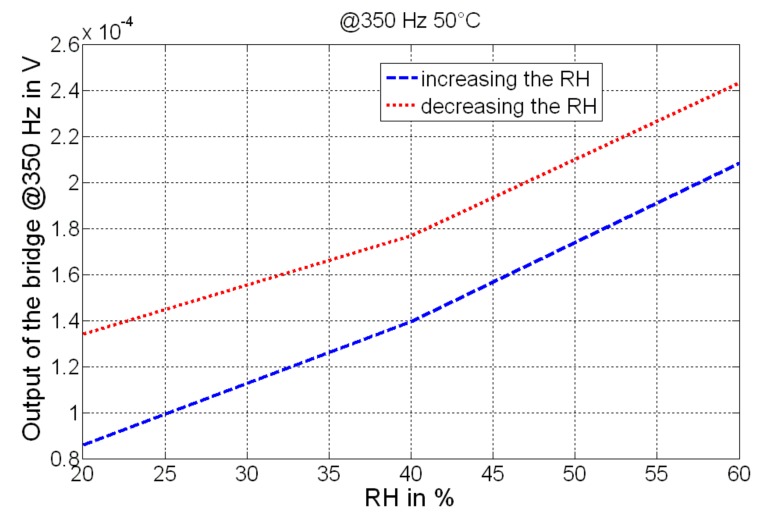
Output of the conditioning circuit used for the MEMS, considering the increasing and the decreasing condition of RH. The analysis has been conducted at a fixed sinusoidal excitation (evaluated at 350 Hz) and at a fixed temperature (50 °C).

**Figure 11:**
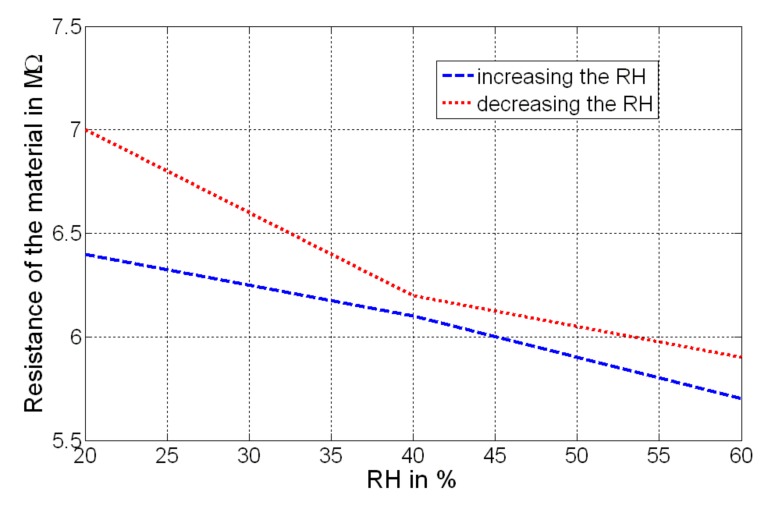
Resistance of the material, considering the increasing and the decreasing condition of RH.

The device realized shows some nonlinear behaviors that have been characterized both in the voltage domain (see Figure 10; output evaluated at 350 Hz) and in the resistance domain (see Figure 11; resistance of the compound). RH was incremented and then decremented, and each condition (value of humidity) was maintained for about 30 min; as can be observed, a hysteretic effect has been detected for both voltage and resistance changes. This represents the output deviation at a certain input signal, when that input is approached first by increasing and then by decreasing the RH. A maximum variation of about 0.4 × 10^−4^ V has been detected at the output of the bridge, while a maximum variation of about 600 kΩ has been measured as the resistance of the GO/PVA.

**Figure 12:**
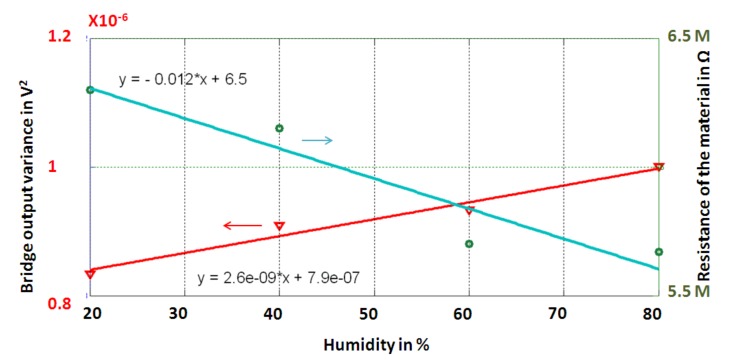
Validation of the redundant principle. It is possible to measure the humidity by using the variation of the output bridge (for a fixed mechanical excitation frequency) and the variation of the resistance of the GO/PVA.

Figure 12 shows the two outputs: the bridge (variance) and the measured resistance of the GO/PVA for several values of humidity. As can be observed, the sensor is capable of measuring the target through the frequency domain and the resistance domain, by using the properties of the GO/PVA. A sensitivity of about −12 kΩ/RH% and ~0.1 Hz/RH% was estimated in the resistance and frequency domain, respectively. Furthermore, a resolution of ~1.8% RH has been experimentally estimated.

## 5. Conclusions

In this work, a hybrid integrated sensor based on graphene oxide/polyvinyl alcohol has been used for measurements of relative humidity. The microsensor has been designed, fabricated, modeled, simulated and an extensive experimental campaign has also been accomplished. In particular, the approach here pursued regards the possibility of performing the measurements by using multiple readouts, with analyses in the frequency and resistance domains. In particular, sensitivities of about 0.1 Hz/RH% and about −12 kΩ/RH% have been estimated. The resolution of the sensor corresponds to ~1.8% RH. Further research will be devoted to demonstrate the effectiveness of the redundant approach, in order to improve immunity to interfering signals with an exhaustive metrological characterization of the MEMS. An optimization step of the microsensor is also a future trend that will be pursued.

## Figures and Tables

**Table 1:** Parameters of the conceived microsensor. The mass has been estimated considering the density of the silicon (2.5 × 10^3^ kg/m^3^) as well as its volume (1600 × 1600 × 450 µm^3^).

*t* [µm]	*w* [µm]	*L_a_* [µm]	*L_b_* [µm]	*k_z_* [N/m]	*M* [kg]	*E_si_* [N/m^2^]	*I* [m^4^]	*ψ* [-]
15	100	800	1600	16.50	2.88 × 10^−6^	1.69 × 10^11^	2.81 × 10^−20^	0.89
